# Early Total Care Versus Damage Control Orthopedics in Floating Knee Injury: Analysis of Radiological and Functional Outcomes

**DOI:** 10.7759/cureus.25615

**Published:** 2022-06-02

**Authors:** Prabhu Ethiraj, Ajay S Shringeri, Arun Prasad P, Arun H Shanthappa, Vishnudharan Nagarajan

**Affiliations:** 1 Department of Orthopaedics, Sri Devaraj Urs Medical College, Sri Devaraj Urs Academy of Higher Education and Research, Kolar, IND

**Keywords:** high-energy trauma, fraser classification, damage control surgery, early total care, floating knee

## Abstract

Introduction

Floating knee injury (FKI) occurs as a result of a high-velocity impact. We assessed the radiological and functional outcomes of FKIs treated by various fixation methods, by damage control orthopedics (DCO) or early total care (ETC).

Materials and methods

We investigated 46 patients with FKI who were operated on between January 2013 and January 2018 at the RL Jalappa Hospital and Research Center, Kolar, India. Functional assessments were evaluated using Karlström and Olerud’s criteria (KOC). Based on their treatments, the patients were divided into the damage control orthopedics group (n = 21) and the ETC group (n = 25). Statistical analyses were used to obtain and compare summary data.

Results

The data of 46 patients were collected. Fractures were classified using the modified Fraser’s classification. Five patients were not included in the final analysis because of death due to complications in the immediate postoperative period. In patients managed by DCO, after radiological union, the functional outcome was excellent in three cases, good in eight, fair in seven, and poor in two. The average time required for radiological union of the femur was 10.75 ± 1.482 months (P = 0.001); for tibia union, it was 10.25 ± 1.682 months (P = 0.011). The average range of knee flexion was 85°± 16.059° (P = 0.001), which was statistically significant. In patients managed by ETC, there were six cases with an excellent functional outcome, 13 with a good outcome, and two with a fair outcome. The average time required for radiological union of the femur was 9.29 ± 1.765 months (P = 0.006); for the tibia, it was 9.05 ± 1.161 months (P = 0.012). The average range of knee flexion was 100° ± 10.954° (P = 0.001), which was statistically significant. Fat embolism was noted in eight cases; four of these patients died due to multiorgan dysfunction. This was the major life-threatening complication in the early definitive fixation group. In the DCO group, only three cases had fat embolism, with one death due to multiorgan dysfunction. Early postoperative infection was a concern in the ETC group, evident in six cases.

Conclusion

The classification system for FKI needs further research, which must include multiple parameters. Fracture classification and patient selection are crucial considerations in deciding the best treatment for a particular fracture.

## Introduction

The concept of floating knee injury (FKI) was proposed by Blake and McBryde in 1975 to describe ipsilateral fractures of the femur and tibia [1]. Typically, FKIs result from high-velocity trauma, such as in road traffic accidents (RTAs) and falls from heights; they are associated with a high incidence of open fractures (59%-67% of cases) [2]. FKIs involve various fractures of the femur and tibia, which were classified by Fraser in 1978 [3].

Fraser’s classification was modified by Ran et al. in 2013. They considered the severity of comminution of articular fractures and associated patellar fractures, as well as their patterns. This modified classification appears to correlate better with outcomes than the original one [4,5]. The incidence of FKI due to RTA is increasing, accounting for about 2.6% of all fractures in 1986 [6]. Many studies have reported that around 50% of FKIs are associated with internal derangement of the knee [7].

Treatment of FKI typically involves early total care (ETC) of the fracture, with the aim of early mobilization of the patient, to promote early healing and reduce the risks of joint stiffness, deep vein thrombosis, and other pulmonary complications [8,9]. The use of definitive fixation techniques can lead to catastrophic, life-threatening complications in a compromised patient as a result of the “second hit” phenomenon. Delaying immobilization increases the odds of mortality. Consequently, damage control orthopedics (DCO) is now replacing ETC [10].

In most situations, therapy should be tailored after weighing the advantages of quick, permanent skeletal stabilization against the potentially fatal dangers of systemic consequences, such as fat embolism, severe lung damage, and multiorgan failure.

DCO is an emergency procedure performed to save the life of a patient. Procedures such as external fixation involve minimally invasive surgery to stabilize major fractures, especially long bones, thereby controlling bleeding.

## Materials and methods

Study design

This study was approved by the Institutional Ethics Committee of Sri Devaraj Urs Medical College (DMC/KLR/IEC/ 632/2021-22). The study included 46 patients who received operations for FKI at RL Jalappa Hospital and Research Center between January 2013 and January 2018. The patient’s details and radiographs were collected from documents available in the hospital’s medical records department.

We included polytrauma patients (aged >20 years) with ipsilateral fractures of the tibia and femur, either closed or open, of Gustilo-Anderson types 1, 2, 3A, and 3B. We excluded type 3C, pathological fractures, associated vascular injury, and crush injury cases. The severity of the injury was assessed, and management plans were decided upon based on the Ganga Hospital Open Injury Score (GHOIS) at presentation.

We included patients who attended follow-up assessments at one, three, six, and 12 months. We collected patients’ details during admission, including GHOIS, mode of injury, and treatment protocol. Clinical and radiological assessments were recorded at each follow-up. Functional assessments were made using Karlström and Olerud’s criteria (KOC) (Table 1) after fracture union was confirmed. We compared our results with those of available standard studies with comparable characteristics. Statistical analyses were performed using IBM SPSS for Windows version 20.0 (IBM Corp., Chicago, IL, USA).

**Table 1:** Karlström and Olerud’s criteria for assessing the outcomes of floating knee injury

	Excellent	Good	Fair/acceptable	Poor
Subjective symptoms of the leg	Nil	Intermittent minimal symptoms	More severe symptoms impairing function	Considerable functional impairment, pain at rest
Subjective symptoms of the knee or ankle	Nil	Intermittent minimal symptoms	More severe symptoms impairing function	Considerable functional impairment, pain at rest
Ability to walk	Unimpaired	Intermittent minimal impairment	Restricted	Uses cane or crutch
Works and sports	Same as before the injury	Given up some sports, work same as before	Change to less strenuous work	Permanent disability
Malangulation, malrotation, or both	Nil	<10°	10°-20°	>20°​​​​​​​
Leg-length discrepancy	Nil	<1 cm	1-3 cm	>3 cm
Restriction of joint movements (hip, knee, and ankle)	Nil	<10°​​​​​​​at the ankle, <20°​​​​​​​ at the hip, knee, or both	10°​​​​​​​-20°​​​​​​​at the ankle, 20°​​​​​​​-40°​​​​​​​ at the hip, knee, or both	>20°​​​​​​​ at the ankle, >40°​​​​​​​ at the hip, knee, or both

## Results

This retrospective study involved 46 patients with FKI. They were predominantly male (n = 43), with a mean age of 41 years. FKI fractures involved the right side in 60.87% of the patients (n = 28). There were 14 open fractures of the femur and 17 of the tibia, with severity ranging from Gustilo-Anderson type 2 to type 3B and GHOIS ranging from 5 to 15. The associated injuries included humerus fractures, distal radius fractures, Monteggia fractures, metatarsal and phalanx fractures, patellar fractures, pubic rami fractures, and degloving injuries involving a leg or foot.

Damage control surgery group

In 21 patients, fractures were initially stabilized with external fixators. Simple extra-articular and stable fractures with adequate soft tissue coverage were fixed with definitive fixation techniques involving less soft tissue insult, such as intramedullary nailing of the tibia or femur, while the fellow long bone across the fracture site received an external fixator. The fixation techniques used were intramedullary interlocking nails for the femur (nine patients) or tibia (two patients), proximal tibia locking compression plates (LCPs) (two patients) using the minimally invasive percutaneous plate osteosynthesis technique, and partial patellectomy and cerclage (two patients with patellar fracture). Soft tissue coverage procedures (e.g., split skin grafting and flap coverage) were performed in 11 cases.

The average time required for femur union was 10.75 ± 1.482 months (P = 0.001); for the tibia, it was 10.25 ± 1.682 months (P = 0.011), which is statistically significant. Seven patients had delayed union of the fracture; hence, the fracture healing process was augmented with bone marrow injection. Joint stiffness was the most common complication observed, with the knee being stiff in nine patients (range of knee flexion: <90°), the hip in six, and the ankle in two. There was a limb length discrepancy noted in six cases and a shortening of 1-2 cm, without any functional impairment. Features of early complications, such as fat embolism, were noted in three patients. Early postoperative infections were noted in four cases, three of which were controlled with antibiotics; however, in one case, the infection persisted until the implant was removed. One patient showed signs of delayed infections at eight months, which subsided after implant removal. Two patients treated using an external fixator and Ilizarov ring fixator developed pin site infections, which subsided with antibiotics and regular pin site dressing. One patient died on the fifth postoperative day due to multiorgan dysfunction following fat embolism.

Upon consideration of radiological union, the patients were asked to bear weight at an average time of five months postoperatively. After radiological union, the assessment of functional outcomes, using Karlström and Olerud’s criteria, showed that three (15%) patients had excellent outcomes, eight (40%) had good outcomes, seven (35%) had fair outcomes, and two (10%) had poor outcomes after surgical management of the FKI using damage control surgery. The average range of knee flexion was 85° ± 16.059° (P = 0.001), which was statistically significant.

Early total care group

A total of 25 patients with borderline hemodynamic stability were operated on using early definitive fixation. The fracture fixation techniques used included intramedullary interlocking nails for the femur in 11 patients and for the tibia in 14 patients. A distal femur locking compression plate (LCP) was used in eight patients and a proximal tibia LCP in 11 patients. Distal femur nailing was performed in six patients. Tension band wiring for patellar fractures was performed in three patients with a simple fracture pattern and cerclage in one patient with comminution of the patella. A bone graft was used in one patient for filling the void created after the reduction of the distal femur articular fracture.

The average time required for radiological union of the femur was 9.29 ± 1.765 months (P = 0.006); for the tibia, it was 9.05 ± 1.161 months (P = 0.012), which was statistically significant. Two patients had delayed union of the fracture; hence, fracture healing was augmented with bone marrow injection. Joint stiffness was observed in two patients, with a range of knee flexion of 80°. Limb shortening of 1-2 cm was observed during the immediate postoperative period in four cases. Early postoperative infections (superficial infections) were noted in six patients, which were managed with serial debridement and parenteral antibiotics, according to the culture and sensitivity. Fat embolism was a serious complication in eight patients; all were treated with low-molecular-weight heparin and supplemental oxygen. Four of these patients died in the immediate postoperative period (postoperative days 3-6) due to multiorgan dysfunction.

**Figure 1:**
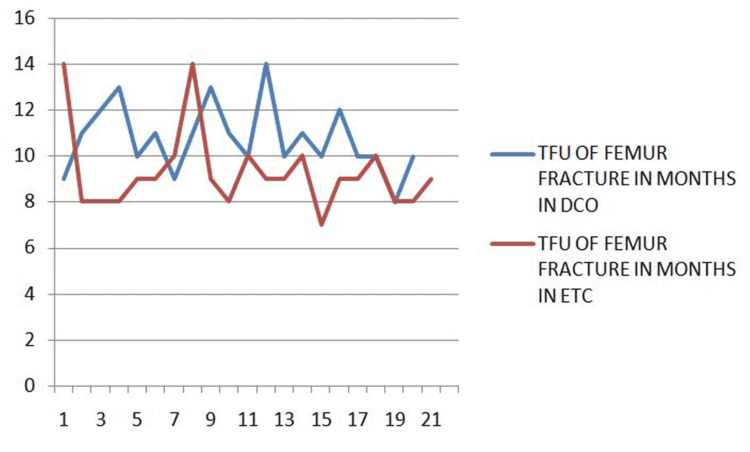
Time for union of femur fracture ETC, early total care; DCO, damage control orthopedics; TFU, time for union

The time for union of femur fracture is compared between the two groups (Figure 1).

**Figure 2:**
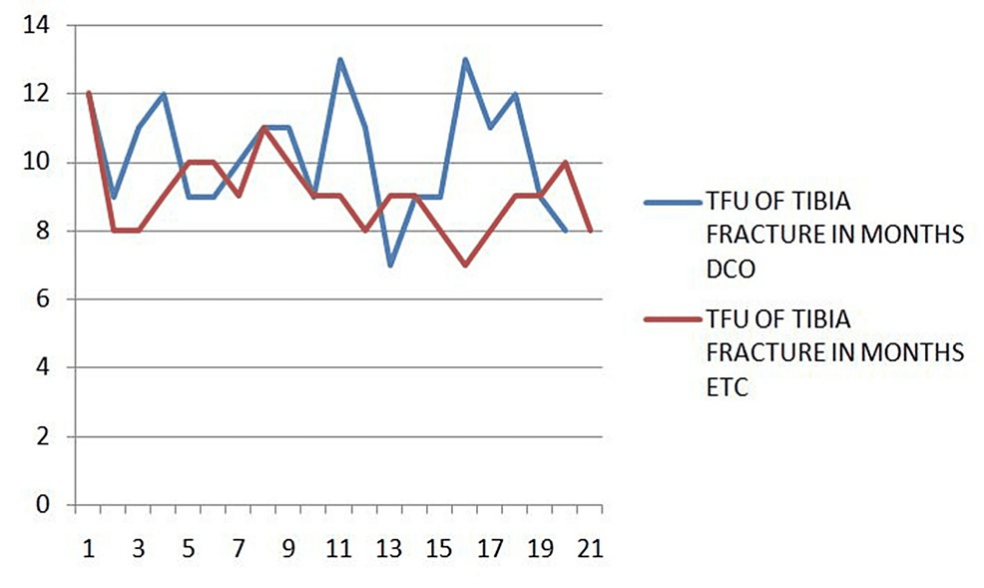
Time for union of tibia fracture ETC, early total care; DCO, damage control orthopedics; TFU, time for union

The time for union of tibia fracture is compared between the two groups (Figure 2).

**Figure 3:**
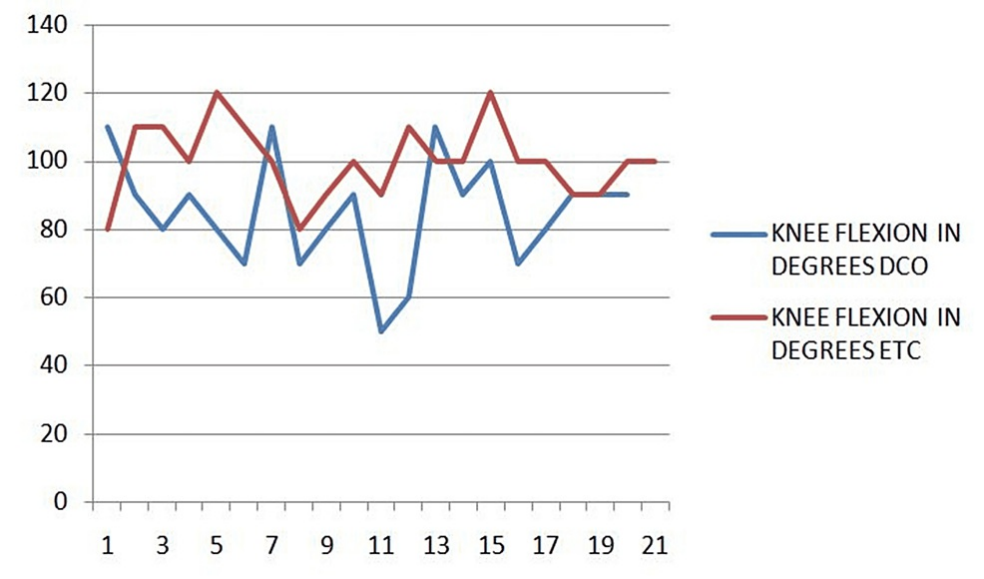
Knee flexion ETC, early total care; DCO, damage control orthopedics

The degree of knee flexion after surgery is compared between the two groups (Figure 3).

**Figure 4:**
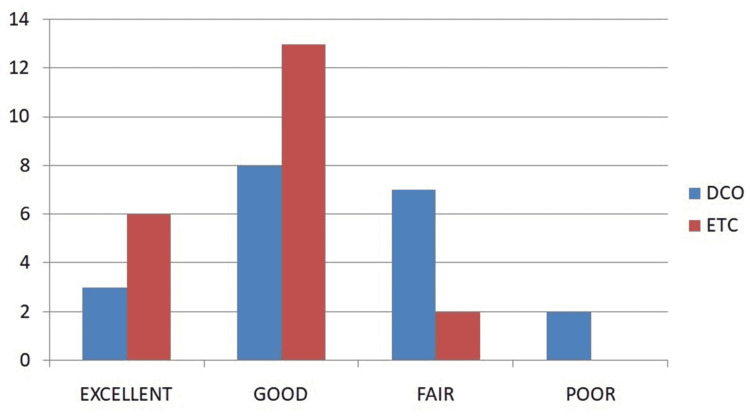
Karlström and Olerud’s outcomes for DCO and ETC ETC, early total care; DCO, damage control orthopedics

In consideration of radiological union, the patients were asked to bear weight at an average time of four months postoperatively. After radiological union, assessments of functional outcomes using Karlström and Olerud’s criteria showed that six patients (28.57%) had excellent outcomes, 13 (62%) had good outcomes, two (9.5%) had fair outcomes, and none had a poor outcome (Figure 4). The average range of knee flexion was 100° ± 10.954° (P = 0.001).

## Discussion

FKIs have become a focus of concern due to their high incidence in the younger generations [2]. A study by Fraser published in 1978 to explain the classification of FKI using 220 cases caused by RTA found that intra-articular fractures had poor outcomes [4]. In our study, the predominant mode of injury was RTA. 

Patients with significant soft tissue injury had greater knee stiffness and a more limited range of knee motion, resulting in poor outcomes.

In our study, the male-to-female ratio was 9:1; the right side-to-left side ratio was 2:1, which corresponds to all of the standard studies. Since FKIs result from high-velocity trauma, the incidence of open injuries is very high. In our study, although there was a high incidence of open fractures of both femur and tibia, only four cases required split skin grafting, and seven cases required flap coverage. Rethnam et al. reported in 2007 that a single-incision technique for fixing both femur and tibia produced satisfactory results [11]. Soft tissue-sparing procedures, such as percutaneous plating, were demonstrated by Lobenhoffer et al. in 1997 [12].

Intramedullary nailing was recommended as a preferred technique for Fraser type 1 fractures by Dwyer et al. in 2005 [13]. In our study, 10 cases were operated upon using a retrograde nail for the femur and intramedullary nailing for the tibia; these cases received much less soft tissue injury due to surgery. Five out of the 10 cases had excellent outcomes, while the others had good outcomes, in terms of early fracture healing and mean range of knee flexion, which was 102° greater than in other methods of fixation.

Range of movement was restored immediately in all bidiaphyseal fractures and after 2-4 weeks in intra-articular fractures. Split skin grafting was performed around 7-10 days after fracture fixation. Fasciocutaneous flap surgery was performed 5-7 days after surgery when the wound was clean and with healthy granulation. For fractures of type 3A in the modified Fraser’s classification, patellar fractures were treated with tension band wiring. Type 3B fractures were managed using partial patellectomy in one case and cerclage in two cases. Patellar fracture has a significant role in functional outcome, hence its inclusion in the modified Fraser’s classification (Table 2) [5].

**Table 2:** The modified Fraser’s classification of floating knee injury

Class	Articular status	Description		Incidence
Type 1	Extra-articular	Bidiaphysis fractures - fractures of both the femur and tibia at the diaphysis		50%
Type 2	Intra-articular	Type 2A	Simple articular	17.4%
		Type 2B	Complex articular	19.6%
Type 3	Associated patellar fracture	Type 3A	Simple patellar fracture	6.5%
		Type 3B	Multifragmentary patellar fracture	6.5%

A previous study recommended that excellent and good outcomes can be regarded as satisfactory results and fair to poor outcomes as unsatisfactory results [14]. In our study, the majority of cases had good to excellent outcomes, while few had fair to poor outcomes.

Better functional outcomes were obtained from early definitive fixation. Patients treated with DCO had fewer life-threatening complications but a greater number of unsatisfactory outcomes. The results of various standard studies on floating knee injury were compared (Table 3).

**Table 3:** Results of various standard studies on floating knee injury

Name of study	Sample size	Excellent	Good	Acceptable/fair	Poor
Fraser et al. (1978) [4]	63	3	15	30	15
Rethnam et al. (2007) [11]	29	15	9	2	3
Aher et al. (2016) [15]	30	3	9	10	8
Schiedts et al. (1996) [16]	18	4	7	-	7
Mohamadean et al. (2017) [17]	21	11	6	3	1
Hee et al. (2009) [18]	89	6	53	25	4
Our study	41	9	21	9	2

Limitations

As a retrospective study, we faced challenges in ascertaining the methods used for initial stabilization, surgical fixation, postoperative management, and wound management, and the precautions to improve union, based only on the documents available. Ligament injuries, in terms of internal derangement of the knee, were not taken into consideration due to the location of the hospital. For improved statistical analyses, further study is required in a larger population.

## Conclusions

FKIs result from high-energy trauma, often in young people, and require extensive surgical planning and rehabilitation. As an aspect of polytrauma, FKI must be ascribed more importance for further research efforts. In our study, we conclude that functional outcomes were better with early definitive fixation but at the cost of increased risk of the “second hit” phenomenon. Damage control surgery posed less risk of mortality but increased the likelihood of delayed union, knee stiffness, and a poor functional outcome. The poor prognostic indicators of FKI are open fractures, intra-articular fractures, initial external fixator application, and soft tissue injury. Hence, a thorough assessment of FKI fracture and judicious management of associated injuries, with proper planning and patient selection for the type of fracture fixation, are important considerations for obtaining good outcomes and reducing complications.
